# Features of wild-type human SOD1 limit interactions with misfolded aggregates of mouse G86R Sod1

**DOI:** 10.1186/1750-1326-8-46

**Published:** 2013-12-17

**Authors:** David A Qualls, Mercedes Prudencio, Brittany LT Roberts, Keith Crosby, Hilda Brown, David R Borchelt

**Affiliations:** 1 From the Department of Neuroscience, Center for Translational Research in Neurodegenerative Disease, SantaFe HealthCare Alzheimer’s Disease Research Center, McKnight Brain Institute, University of Florida, Box 100159, 1275 Center Drive, Room J491, Gainesville, FL 32610, USA; 2 Department of Neuroscience, Mayo Clinic Jacksonville, 4500 San Pablo Rd S, Jacksonville, FL 32224, USA

## Abstract

Mutations in the gene encoding superoxide dismutase 1 (SOD1) account for about 20% of the cases of familial amyotrophic lateral sclerosis (fALS). It is well established that mutations in SOD1, associated with fALS, heighten the propensity of the protein to misfold and aggregate. Although aggregation appears to be a factor in the toxicity of mutant SOD1s, the precise nature of this toxicity has not been elucidated. A number of other studies have now firmly established that raising the levels of wild-type (WT) human SOD1 (hSOD1) proteins can in some manner augment the toxicity of mutant hSOD1 proteins. However, a recent study demonstrated that raising the levels of WT-hSOD1 did not affect disease in mice that harbor a mouse *Sod1* gene (*mSod1*) encoding a well characterized fALS mutation (G86R). In the present study, we sought a potential explanation for the differing effects with WT-hSOD1 on the toxicity of mutant hSOD1 versus mutant *mSod1*. In the cell culture models used here, we observe poor interactions between WT-hSOD1 and misfolded G86R-mSod1, possibly explaining why over-expression of WT-hSOD1 does not synergize with mutant mSod1 to accelerate the course of the disease in mice.

## Introduction

Mutations in the gene encoding superoxide dismutase 1 (SOD1) account for about 20% of the cases of familial amyotrophic lateral sclerosis (fALS) {http://alsod.iop.kcl.ac.uk/default.aspx}. The SOD1 protein is a relatively small antioxidant enzyme comprised of 153 amino acids. In its active state, the protein dimerizes to form the mature enzyme with each subunit binding 1 atom of Zn and 1 atom of Cu [1-3]. To date more than 165 mutations in more than half of the amino acid residues in the enzyme have been identified in patients diagnosed with fALS {http://alsod.iop.kcl.ac.uk/default.aspx}. The impact of these mutations on its enzymatic activity varies greatly, and it has not yet been possible to define a single mechanism by which these mutations cause disease [for review see [4]. One common feature of mutant SOD1 proteins is that they exhibit a high tendency to aggregate aberrantly into high molecular weight structures, which can be isolated biochemically due to their insolubility in non-ionic detergents [5].

Although aggregation of mutant SOD1 is by some manner linked to the toxic property of the protein that is responsible for inducing motor neuron disease, the nature of the toxic property remains incompletely defined and may be multifactorial [4]. One of the more interesting avenues towards improving our understanding of the basis of mutant SOD1 toxicity has been revealed in studies in which mice expressing mutant SOD1 have been mated to mice expressing wild-type hSOD1. A number of studies have now firmly established that raising the levels of WT-hSOD1 proteins by crossing a strain of mice produced by Gurney et al. (Gur WT [6]) to mutant hSOD1 mice can, in some manner, accelerate the onset of paralysis [7-11]. Importantly, the Gur WT mice express WT-hSOD1 at very high levels and lines of WT-hSOD1 mice that express at lower levels do not as uniformly accelerate disease [11,12].

The basis for the current work is a recent study by Audet and colleagues that asked whether overexpression of WT-hSOD1, using the Gur WT strain, could augment the toxicity of G86R-mSod1, finding no effect [13]. The G86R mouse [14], was created by a similar approach to what has been used for mice that express mutant human proteins; a genomic DNA fragment of the mouse gene was mutated to encode an fALS mutation. The G86R mutation in mouse Sod1 is equivalent to the human G85R hSOD1 mutation. The difference in numbering is a consequence of the historical numbering of human SOD1 residues based on the mature human protein sequence (initiator methionine removed post-translationally) whereas the mouse sequence was numbered by actual codon position. The lack of an effect of co-expressed WT-hSOD1 on disease in the G86R mice is not a peculiarity of the mutation because mice generated by crossing Gur WT animals to mice expressing G85R-hSOD1 develop motor neuron disease considerably earlier than mice expressing the mutant protein alone [10]. The different outcomes in these two experimental settings implies that improving our understanding of the way WT-hSOD1 interacts with G85R hSOD1 and G86R-mSod1 could provide insight into how the presence of WT-hSOD1 accelerates the onset of paralysis.

To study interactions between hSOD1 and mSod1 that may occur when fALS mutations induce misfolding, we have employed an established cell model of mutant SOD1 aggregation in conjunction with co-transfection approaches. Using a detergent extraction and sedimentation assay, we examine whether WT-hSOD1 interacts with misfolded G86R-mSod1. Additionally, to visualize such interactions, and following a strategy previously described [11,15], we have fused the SOD1 variants to fluorescent tags; G86R-mSod1 fused to red fluorescent protein (RFP) and WT-hSOD1 or WT-mSod1 fused yellow fluorescent protein (YFP). Using both biochemical and visual methods we compare the degree to which WT-mSod1 and WT-hSOD1 interact with misfolded aggregates of G86R-mSod1.

## Methods

### Generation of hSOD1/mSod1 DNA expression plasmids and split luciferase constructs

Expression plasmids (pEF.Bos [16]) that encode wild-type (WT) hSOD1, WT-mSod1, A4V-hSOD1, and G85R-hSOD1 have been previously described [17-19]. The G86R mutation in mSod1 was introduced into pEF. Bos-WT-mSod1 using oligonucleotides that encode the desired mutation and the Quick Change mutagenesis kit (Stratagene/Life Technologies/Thermo, Grand Island, NY). WT-hSOD1mon and WT-mSod1mon were created by introducing mutations at codons 50 and 51 to change amino acids F and G at these positions to E. Mutations were introduced in pEF.Bos vectors for WT-hSOD1 and WT-mSod1 using oligonucleotides encoding the desired mutations and the Quick Change Kit. pEF.Bos vectors encoding WT-hSOD1 and G85R-hSOD1 fused to turboRFP (Evrogen, Moscow, Russia) and eYFP (Invitrogen/Life Technologies/Thermo Grand Island, NY,) have also been previously described [11,15]. To create pEF.Bos expression plasmids that encode fusions of G85R-hSOD1 and G86R-mSod1 to YFP and RFP, and WT-hSOD1mon and WT-mSod1mon fused to YFP and RFP, we started with pEF.Bos vectors encoding WT-hSOD1:YFP or WT-hSOD1:RFP fusions and removed the segment of the vector encoding SOD1 by digestion with unique (engineered) Nco 1 and Sal 1 restriction endonuclease sites. We then inserted cDNA encoding, WT-mSod1, G86R-mSod1, WT-hSOD1mon and WT-mSod1mon engineered with compatible ends to produce the new vectors encoding in-frame fusion proteins. All vectors were extensively sequenced to verify the presence of the desired mutations and the absence of any unwanted mutations.

The split, humanized, Gaussia luciferase (hGluc) constructs (New England Biolabs, Ipswich, MA, USA) were generated by cleaving pEF.Bos vectors encoding WT-hSOD1 or WT-hSOD1mon with Sal1 and inserting segments of cDNA encoding hGluc in-frame at the C-terminus of the SOD1 cDNAs. Each hGluc segment was amplified using standard PCR procedures with the primers hGluc-S (cgcagctgcagtcgaccgatggtggcggtggctct) and hGluc1-BOS-AS (tcccaggtgggtaccttagcctatgccgccctgt) for the N-terminal segment (L1) or hGluc2-BOS-AS (tcccaggtgggtaccttagtcaccaccggcccc) for the C-terminal segment (L2) of luciferase. PCR products were analyzed on a 1% agarose gel to confirm the target fragment was amplified. In most cases a single PCR product was present. If there were multiple PCR products, the desired fragment was cut from a preparative agarose gel, extracted, and purified using the QIAquick Gel Extraction kit (Qiagen, Germantown, MD, USA; Cat. No 28706). PCR products were inserted into the cleaved pEF.Bos-SOD1 vectors using the In-Fusion Cloning kit (Clontech, Mountain View, CA; Cat. No 639601). All plasmids were then transformed into Stellar competent cells (Clontech) following standard transformation strategies. The FastPlasmid Mini Kit (5 Prime, Gaithersburg, MD, USA; Ref# 2300010) was used to isolate new recombinants, which were verified by DNA sequence analysis. Upon verification of the correct plasmid DNAs, large scale preparations of the different plasmids for transfection were prepared by standard cesium chloride gradient purification.

### Transient transfections and microscopy analyses

Expression of hSOD1/mSod1 constructs for analyses of detergent-insolubility were performed in HEK293FT cells as explained in figure legends and following procedures described in previous studies [5,20]. Briefly, the expression plasmids were transiently transfected into HEK293FT cells. Following the times indicated (24 or 48 hrs), the cells were harvested and subjected to detergent extraction and sedimentation to separate soluble and insoluble forms of SOD1. Detergent fractions were analyzed by SDS-PAGE and immunoblot with SOD1 antibodies. Immunoreactive proteins were visualized and quantified on a Fuji Imaging system. Each experiment was repeated and quantified at least 3 times.

For studies to visualize inclusions formed by YFP and RFP fusion constructs, we transfected Chinese Hamster Ovary (CHO) cells because these cells normally show a very flat morphology with a distinct nucleus and cytoplasm; allowing for a good visualization of intracellular inclusions. These cells also show good adherence to culture plates and resist lifting after saponin treatment. Cells were split into 12-well plates containing Poly-L-Lysine coated coverslips, and incubated at 37°C with 5% CO_2_ for 24 hours. Cells were transiently transfected with the vectors of interest using Lipofectamine-2000 (single transfections: 500 ng total DNA used; Co-transfections: 500 ng of each construct used). Twenty-four hours after transfection, one set of cells were treated with 0.1% saponin in 1x PBS for 30 minutes. The cells were then rinsed with 1x PBS, and fixed in 4% paraformaldehyde (1x PBS). A 1:2000 solution of DAPI in 1x PBS was used to stain nuclei. Coverslips were then mounted on slides for analysis via fluorescence microscopy. All experiments were performed three times, and each sample was analyzed for the presence and composition of inclusion-like structures. Representative examples of cells from each sample were photographed. The camera exposures used to capture RFP and YFP images in co-transfections were recorded and compared to single transfections to ensure that the fluorescence was the result of the YFP, and not bleed-through from co-expressed RFP.

For each construct analyzed, multiple transfections were performed (at least 3) and multiple images for each transfection experiment were captured. Between 250 and 1,000 cells expressing the fluorescent fusion protein were assessed for each experiment, depending upon the transfection efficiency for a particular experiment. The images shown in the relevant figures are representative of at least 3 individual experiments.

### Luciferase assay

CHO cells were cultured into 6 well plates and transiently transfected, at 90-95% confluency, with equimolar amounts of two split luciferase constructs (total 2 μg DNA, 1 μg per construct). Co-transfections were performed using Lipofectamine 2000, following the manufacturer’s protocol (Invitrogen, Carlsbad, CA, USA). The cells were incubated at 37°C in a CO_2_ incubator for 24 hours, rinsed with 1x PBS, harvested, and the cells were pelleted by centrifuging at 5000 xg for 2 min. A coelenterazine assay was used in order to demonstrate the luciferase activity of the split luciferase constructs. The cell pellets were resuspended in approximately 5x estimated pellet volume in 1x PBS containing protease inhibitor cocktail (Sigma, St. Louis, MO; cat #P8340) and lysed by three freeze-thaw cycles in a dry-ice/100% alcohol solution and a 42°C water bath. The samples were centrifuged and the supernatant was transferred to new Eppendorf tubes. The supernatant was diluted 1:20 and 2 μl of each diluted sample was transferred to a 96-well plate. The coelenterazine assay was performed following the manufacturer’s protocol (Nanolight Technology, Pinetop, AZ, USA; Cat. No 303–500) using a microplate reader (Synergy HT, Biotech instruments). This assay was repeated two more times with each repeated transfection.

### Statistical analyses

Data on relative aggregation propensity were analyzed on GraphPad PRISM 5.01 Software (La Jolla, CA) to determine statistical differences, using unpaired student *t*-tests, and generate graphic representations.

## Results

### WT-hSOD1 does not readily co-aggregate with G86R-mSod1

**Figure 1:**
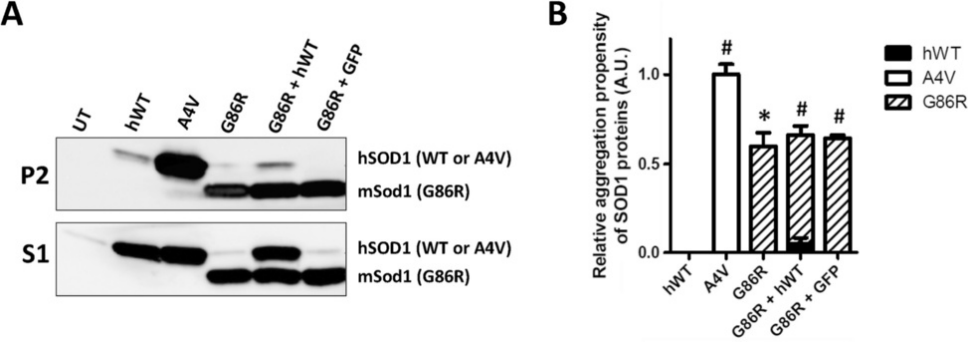
**WT-hSOD1 does not co-sediment with aggregates formed by G86R-mSOD1. A)** Immunoblot of detergent-insoluble (P2) and soluble (S1) fractions of HEK293FT cells transfected for 48 hours with vectors for WT-hSOD1 (hWT), A4V-hSOD1 (A4V), G86R-mSod1 (G86R), or co-transfected with 2 vectors (hWT + G86R and GFP + G86R). The GFP vector served as a co-transfection control. Note that G86R-mSod1 migrates faster than the hSOD1 constructs as noted on right side of the Figure. **B)** Quantification of the relative aggregation propensity of each transfected protein. Paired student *t*-tests were performed to establish significant differences with hWT: *p ≤ 0.05, ^#^*p* ≤ 0.005. Bars represent mean ± SEM. Note that the aggregation propensity of WT-hSOD1 in cells co-transfected with G86R-mSod1 is not significantly greater than when expressed alone.

In order to determine whether WT-hSOD1 co-aggregates with or otherwise modulates aggregation of G86R-mSod1, we used a cell culture model of mutant SOD1 aggregation that we have used extensively in the past. Human embryonic kidney cells (HEK293FT) were transiently transfected with the indicated mouse and human SOD1 constructs. Forty-eight hours following transfection, cells were harvested and detergent-soluble (S1) and detergent-insoluble (P2) fractions were obtained as previously described [19]. Note that the aggregation propensity of SOD1 proteins is measured by the ratio of detergent-insoluble to detergent-soluble SOD1. A common characteristic for both WT-hSOD1 and WT-mSod1 proteins is their low inherent aggregation propensity when expressed in cultured cells [17,19]. As expected, expression of WT-hSOD1 in our cell culture system induced little aggregation of this protein as assayed by the formation of detergent insoluble complexes (hWT, Figure 1A and B). In each experiment, expression of the A4V variant of hSOD1 provided a positive control, which robustly aggregated (A4V, Figure 1A and B). Compared to WT-hSOD1, G86R-mSod1 showed a much higher tendency to aggregate (Figure 1A, P2 upper panel; and B). At 48 hours post-transfection, cells co-expressing WT-hSOD1 with G86R-mSod1 showed no statistically significant decrease in the level of detergent insoluble mutant mSod1 nor did we observe a statistically significant increase in the level of insoluble WT-hSOD1 in cells expressing both proteins (Figure 1A and B). These data suggest that WT-hSOD1 does not readily interact with misfolded G86R-mSod1 that is organized into detergent-insoluble complexes.

### Visualization of hSOD1/mSod1 inclusion formation

**Figure 2:**
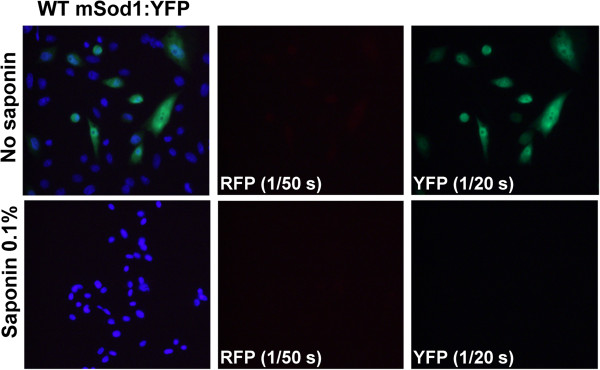
**Fusions of WT-mSod1 with YFP are fully soluble.** WT-mSod1:YFP proteins were expressed in CHO cells for 24 h. Cells were fixed directly or treated with 0.1% Saponin prior fixation. Cells were immunostained with DAPI to allow visualization of cells nuclei. Note the camera exposure times used to capture YFP images were recorded as indicated in the figure. For this figure and the relevant figures that follow, the images shown are representative of 3 independent experiments.

**Figure 3:**
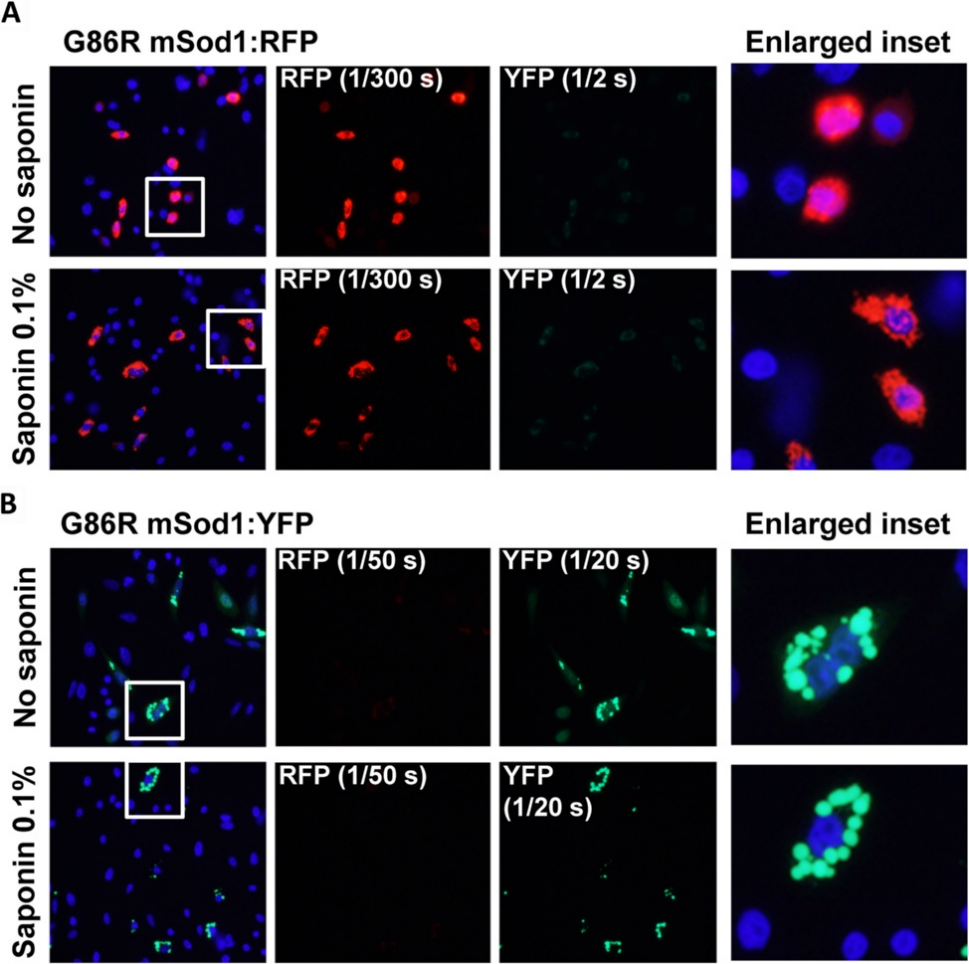
**G86R-mSod1 fused with either RFP or YFP forms saponin-resistant inclusions. (A)** G86R-mSod1:RFP and **(B)** G86R-mSod1:YFP proteins were expressed in CHO cells for 24 h. Cells were fixed directly or treated with 0.1% Saponin prior fixation. Cells were immunostained with DAPI to allow visualization of cells nuclei. Note the camera exposure times used to capture RFP and YFP images were recorded as indicated in the figure. Digitally enlarged insets are shown to the right.

Previous characterization of WT-hSOD1 fused to green fluorescent protein (GFP) have demonstrated normal dimerization and full activity of the SOD1 protein, and thus fusion of SOD1 with such fluorescent reporters does not necessarily alter the folding of SOD1 [21]. We have previously used a strategy in which WT and mutant SOD1 was fused to either red fluorescent protein (RFP) or yellow fluorescent protein (YFP) as a means to observe interactions between the WT and mutant proteins within aggregates [11,15]. We have also developed a strategy in which cells are treated with saponin prior to fixation to distinguish soluble, or diffusible, molecules from insoluble, or immobile, molecules. Saponin is an amphipathic glycoside that creates holes in the plasma membrane without lysing the cell [for review see [22]. Fusions of WT-hSOD1 to YFP are diffusely distributed throughout the cytosol and readily diffuse out of cells permeabilized with saponin; properties consistent with fully soluble proteins [15,23]. Unlike the GFP and YFP tags, the RFP tag has a major impact on the behavior of WT-hSOD1, causing the protein to form a single large round inclusion-like structure in the cytosol that does not diffuse out of permeabilized cells [15]. Similarly, we observed that WT-mSod1 fused to RFP also produced inclusions that were saponin resistant whether expressed alone or in combination with other YFP tagged proteins (Additional file 1: Figures S1 and S2). We have more recently determined that WT-hSOD1:RFP forms inclusions because RFP has a high propensity to dimerize and when paired with WT-hSOD1 the fusion molecule becomes bivalent with an ability to form networks of interactions between molecules [23]. However, when RFP is fused to mutant hSOD1, then the inherent propensity of the mutant hSOD1 to aggregate becomes the primary force in behavior and inclusions formed by mutant SOD1 fused to RFP produce multiple small inclusions that ring the nucleus; these inclusions are identical in morphology to inclusions formed by mutant SOD1 fused to YFP [23]. Thus, in the present study, we used a paradigm in which G86R-mSod1 was fused to RFP and co-expressed with WT-hSOD1 or WT-mSod1 fused to YFP. Similar to WT-hSOD1, WT-mSod1 fused to YFP showed a diffuse distribution and all of the fluorescence was released by saponin treatment (Figure 2). Similar to G85R-hSOD1 [23], G86R-mSod1 fused to either RFP or YFP formed multiple small inclusions that were located near the nucleus and resistant to release by saponin (Figure 3). An important element in these studies to note is that the RFP protein is much brighter than the YFP protein and thus the exposure times were adjusted to capture the images at equivalent intensities. Typically, images of RFP fluorescence were captured with exposures of 1/200 to 1/300 seconds whereas exposures of YFP fluorescence were typically 1/20 to 1/30 seconds; extended to up to 1/2 to 1/3 seconds in some cases. We observed that exposure times of up to 1/2 to 1/3 seconds in the YFP channel were possible, with minimal bled-through of RFP into the YFP channel (see Figure 3A column 3, row 2). Collectively, these findings indicate that WT and mutant mSod1 behave similarly to WT and mutant hSOD1 in terms of propensity to aggregate.

**Figure 4:**
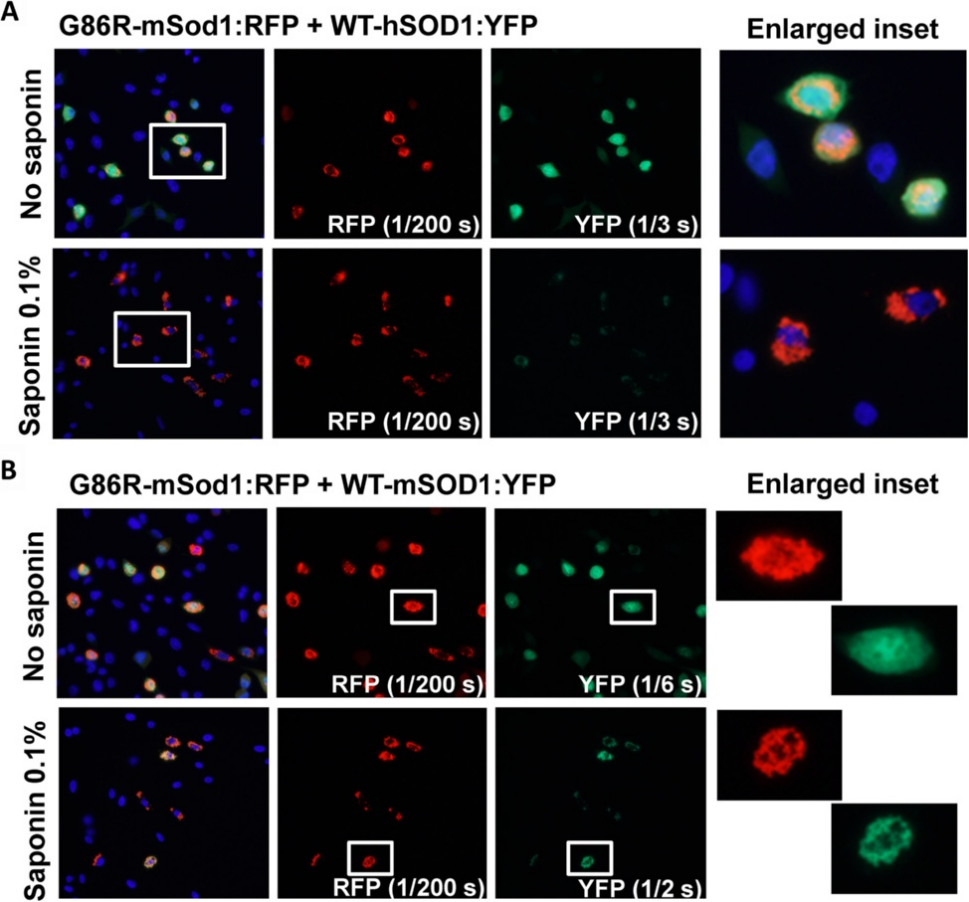
**Co-transfection of mutant mSod1 fused to RFP with YFP fusions of WT-mSod1 and WT-hSOD1. (A** and **B)** The indicated fused constructs were expressed in CHO cells as explained in previous figure legends and Methods. Representative images of 3 independent experiments are shown along with digitally enlarged insets to the right.

**Figure 5:**
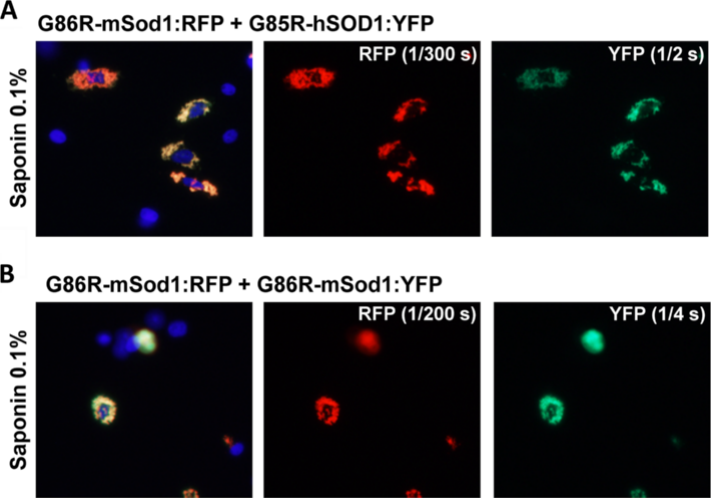
**Co-transfection of mutant mSod1 fused to RFP with G85R-hSOD1:YFP.** The indicated fused constructs were expressed in CHO cells as explained in previous figure legends and Methods. **A)** Co-transfection of vectors for G86R-mSod1:RFP with G85R-hSOD1:YFP. **B)** Co-transfection of vectors for G86R-mSod1:RFP with G86R-mSod1:YFP.

In co-transfection experiments, we observed that inclusions formed by G86R-mSod1:RFP contained little if any WT-hSOD1:YFP within the aggregates that remained after saponin treatment (Figure 4A). By contrast, WT-mSod1:YFP appeared to be tightly bound to G85R-mSod1:RFP inclusion (Figure 4B). To assess whether human and mouse SOD1 were by some manner unable to form intermingled inclusions, we co-expressed G86R-mSod1:RFP with G85R-hSOD1:YFP, finding that the mutant human protein formed co-mingled inclusions with the mutant mouse protein (Figure 5A). The degree of co-mingling was similar to what was observed when G86R-mSod1:RFP was co-expressed with G86R-mSod1:YFP (Figure 5B). Notably, in our assessment of the images we generally observed an all or none response; meaning that in cells in which both proteins were over-expressed either all of protein co-localized or none co-localized. Although such data are easily quantified, the outcome does not produce a range of values and thus it was unnecessary to tabulate quantified data (Table 1 provides a summary of the observed data). In the context of these observations, the weak interaction between WT-hSOD1:YFP and G86R-mSod1:RFP is indicative of some degree of incompatibility between these proteins in generating aggregates.

**Table 1:** Comparison of assays for interactions between WT and misfolded mutant SOD1

**Construct**	**Detergent insoluble**	**Saponin-resistant RFP or YFP tagged inclusions**	
WT-hSOD1	No	WT-hSOD1:YFP	No^1^
		WT-hSOD1:RFP	Yes^1^
WT-hSOD1mon	Yes	WT-hSOD1mon:YFP	No^2^
		WT-hSOD1mon:RFP	No^2^
WT-mSod1	No^1^	WT-mSod1:YFP	No
		WT-mSod1:RFP	Yes^3^
WT-mSod1mon	Yes	WT-mSod1mon:YFP	Yes
		WT-mSod1mon:RFP	Yes
G86R-mSod1	Yes	G86R-mSod1:YFP	Yes
		G86R-mSod1:RFP	Yes
WT-mSod1	?	WT-mSod1:YFP	Yes
+		+	
G86R-mSod1	Yes 48 hr	G86R-mSod1:RFP	Yes
WT-mSod1mon	Yes 48 hr	WT-mSod1mon:YFP	Yes
+		+	
G86R-mSod1	Yes 48 hr	G86R-mSod1:RFP	Yes
WT-hSOD1	No 48 hr	WT-hSOD1:YFP	No
+		+	
G86R-mSod1	Yes 48 hr	G86R-mSod1:RFP	Yes
WT-hSOD1mon	Yes 48 hr	WT-hSOD1mon:YFP	No
+		+	
G86R-mSod1	Reduced 48 hr	G86R mSod1:RFP	Yes
G85R-hSOD1	Yes	G85R-hSOD1:YFP	Yes^2^
		G85R-hSOD1:RFP	Yes^2^
WT-hSOD1	Yes 48 hr^1^	WT-hSOD1:YFP	No^2^
+		+	
G85R-hSOD1	Yes 48 hr^1^	G85R-hSOD1:RFP	Yes^2^
WT-hSOD1mon	Yes 48 hr	WT-hSOD1mon:YFP	No^2^
+		+	
G85R-hSOD1	Yes 48 hr	G85R-hSOD1:RFP	Yes^2^
WT-mSod1	?	WT-mSod1:YFP	No^4^
+		+	
G85R-hSOD1	Yes 48 hr	G85R-hSOD1:RFP	Yes^4^
WT-mSod1mon	?	WT-mSod1mon:YFP	Yes^5^
+		+	
G85R-hSOD1	Yes 48 hr	G85R-hSOD1:RFP	Yes^5^

^1^data from Prudencio et al. [19]; ^2^data from Qualls et al. [23]; ^3^Additional file 1: Figure S1; ^4^Additional file 1: Figure S4; ^5^Additional file 1: Figure S6.

### Role of normal dimeric interactions in mutant SOD1 aggregation

**Figure 6:**
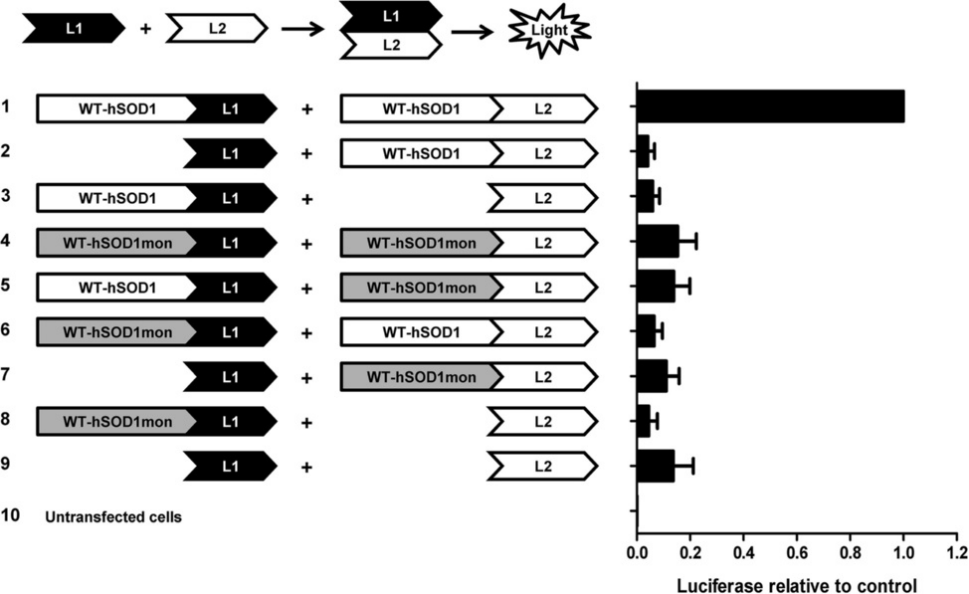
**Mutations at residues F50E/G51E of SOD1 produces a variant of SOD1 that is unable to dimerize with itself or heterodimerize with native WT-hSOD1 subunits.** CHO cells were transiently transfected with indicated split luciferase constructs (illustration) and as explained in Methods. A coelenterazine assay was used in order to demonstrate the luciferase activity of the split luciferase constructs. Each assay was repeated at least 3 times and luminescence values were quantified. For each independent experiment, the values were normalized to the value obtained when fusions of each segment of Gluc fused to WT-hSOD1 were co-transfected.

Misfolded/aggregated forms of mutant SOD1 selectively react with antibodies raised against sequences that are normally inaccessible due to location within the dimer interface [15,24]. To determine whether disrupting the dimerization of WT-hSOD1 or WT-mSod1 may affect interaction with misfolded mutant SOD1, we utilized experimental mutations known to monomerize SOD1 (SOD1-F50E/G51E; [25,26]) . To confirm that the WT monomers of SOD1 (termed SOD1mon) can no longer form dimeric interactions with native SOD1, we generated a series of split-luciferase reporter constructs [27] in which the WT monomer of hSOD1 (WT-hSOD1mon) or native WT-SOD1 was fused to the N-terminal or C-terminal half of humanized Gaussia luciferase (Figure 6). As expected, co-expression of WT-SOD1 fused to the N-terminal half of luciferase (L1) with WT-SOD1 fused to the C-terminal half of luciferase (L2) produced high levels of luciferase activity as the homodimerization of WT-SOD1 brought the two domains of luciferase in close enough proximity to reconstitute enzymatic activity (Figure 6). Also as expected, when WT-hSOD1mon was fused to L1 and L2 domains and co-transfected, then much less luciferase activity was detected (Figure 6). For this latter pair of constructs, the level of activity was similar to what was detected in cells expressing the two domains of luciferase alone (Figure 6; L1/L2). Similarly, very low levels of activity were detected by any combination of WT-hSOD1mon and WT-hSOD1 fusions to L1 or L2 (Figure 6). Immunoblots of cells transfected with the same constructs used for luciferase assay demonstrated relatively equal expression of the WT-hSOD1 and WT-hSOD1mon fusion constructs (Additional file 1: Figure S3). These findings indicate that the introduction of the EE mutations at residues 50/51 of SOD1 produces a variant of SOD1 that is unable to homodimerize with itself or heterodimerize with native WT-SOD1.

**Figure 7:**
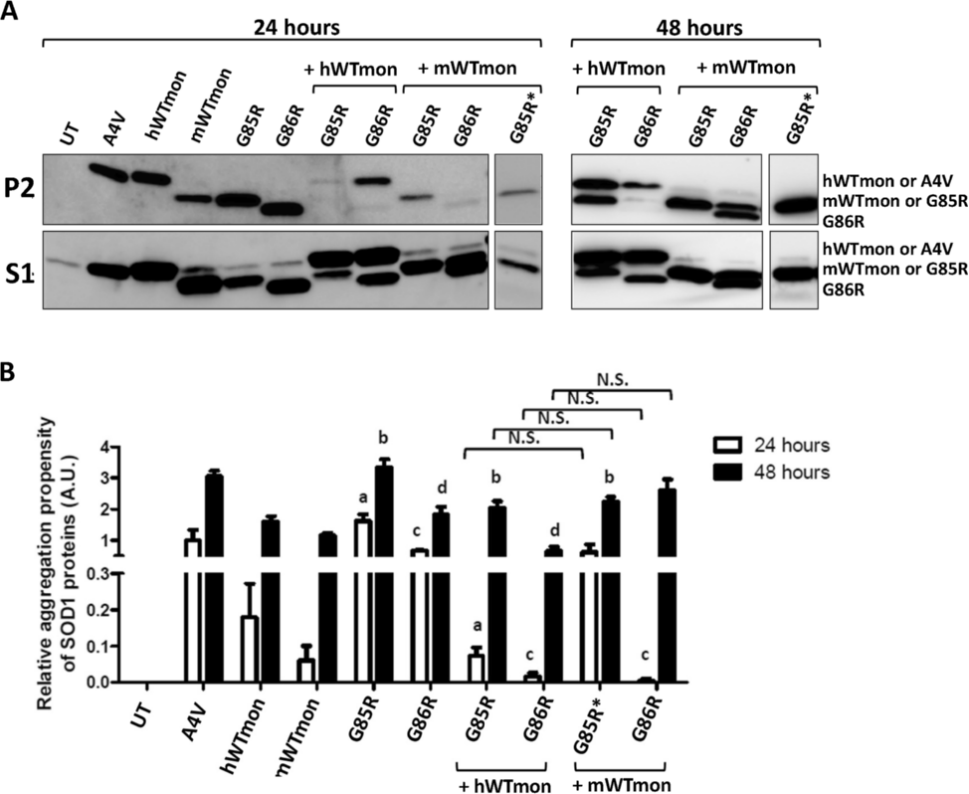
**The introduction of experimental mutations that monomerize WT-SOD1 produce varied effects on interactions with mutant SOD1. A)** Immunoblots of detergent-insoluble (P2) and soluble (S1) fractions of HEK293FT cells transfected with mouse and/or human SOD1 constructs for 24 (left panels) or 48 (right panels) hours. Blots were probed with an antibody that recognizes both mouse and human SOD1 proteins. Asterisk denotes probing with an antibody that is specific against human SOD1 protein. **B)** Quantification of the relative aggregation propensity, as described in Figure 1, for cells transfected for 24 (black bars) or 48 (white bars) hours. Statistical analyses was performed to establish whether the measured amount of insoluble mutant SOD1 in cells expressing only G85R-hSOD1, or only G86R-mSod1, differed from the amount that became insoluble when either was co-expressed with monomerized variants of mSod1 and hSOD1. Explanations for notations on the graph are as follows. The levels of insoluble G85R-hSOD1 in cells expressing only the mutant SOD1 were significantly (*p* ≤ 0.05) higher than in cells co-transfected with WT-hSOD1mon (hWTmon) at 24 hrs (a) and 48 hrs (b) or WT-mSod1mon (mWTmon) at 48 hrs (b). The level of G86R-mSod1 in cells expressing only mutant SOD1 were significantly (*p* ≤ 0.05) higher than in cells co-transfected with WT-hSOD1mon or WT-mSod1mon at 24 hrs (c) or WT-hSOD1 at 48 hrs (d). Bars represent mean ± SEM.

We examined how co-expression of the monomer versions of WT-hSOD1 and mSod1 affected the aggregation of G85R-hSOD1 and G86R-mSod1 in co-transfection studies. Interestingly, compared to native WT, we found that monomeric WT mouse and human SOD1 proteins were highly prone to form detergent insoluble complexes (Figure 7A, P2 fraction; aggregation index similar to A4V-hSOD1 at 48 hours). When the WT monomer of hSOD1 (WT-hSOD1mon) was co-expressed with G85R-hSOD1, we observed a decrease in the levels of insoluble mutant SOD1 that accumulated in 24 hours (Figure 7A). This outcome was similar to what we previously observed with native WT-hSOD1 when co-expressed with G85R-hSOD1 [19]. Interestingly, in these co-transfections, the levels of highly insoluble WT-hSOD1mon were also significantly decreased when co-expressed with G85R-hSOD1 (Figure 7A). Co-expression of WT-hSOD1mon with the G86R-mSod1 mutant for 24 h also significantly reduced the aggregation of the ALS mutant (Figure 7A); however, in this case, a significant accumulation of insoluble WT-hSOD1mon remained detectable (Figure 7A). Co-expression of WT-mSod1mon with G85R-hSOD1 significantly lowered the accumulation of insoluble mutant hSOD1 over 24 hour periods (Figure 7A; lane marked with an asterisk was probed with an antibody specific to hSOD1). Co-expression of WT-mSod1mon with G86R-mSod1 also resulted in diminished accumulation of mutant mSod1 with a coincident reduction in the accumulation of insoluble WT-mSod1mon (Figure 7A). Thus, in these experiments, which assay the formation of detergent-insoluble complexes, we observe that a variant of WT-SOD1 that lacks the normal dimer interface retains the ability to modulate the aggregation of either mouse or human mutant SOD1. In this regard, the monomerized version of WT-SOD1 from either species is similar to what we have previously observed for native versions of these proteins [19].

To assess whether monomerized versions of WT-SOD1 may ultimately co-aggregate with mutant SOD1, we extended the interval post-transfection to 48 hours before harvesting cells for detergent fractionation. By 48 hours post-transfection, co-sedimentation of detergent-insoluble WT-hSOD1mon with G85R-hSOD1 was observed (Figure 7A); similar to what we had previously observed when native WT-hSOD1 is co-expressed with G85R-hSOD1 [19]. Interestingly, in cells co-expressing WT-hSOD1mon with G86R-mSod1 we observed a persistent suppression of mutant mSod1 aggregation (Figure 7A). Thus, even though WT-hSOD1mon has a higher inherent propensity to form detergent-insoluble complexes than native WT-hSOD1, and it is unable to dimerize, WT-hSOD1mon showed a far greater ability to slow the aggregation of G86R-mSod1 than native WT-hSOD1 (compare Figure 1A with Figure 7A). In the reciprocal experiment, when WT-mSod1mon was co-transfected with G85R-hSOD1 for 48 hours post-transfection, we observed that co-expression of WT-mSod1mon no longer inhibited aggregation of G85R-hSOD1 (Figure 7A; lane marked with an asterisk was probed with an antibody specific to hSOD1). When WT-mSod1mon was co-expressed with G86R-mSod1, then both proteins could be detected in detergent-insoluble fractions, similar to when WT-hSOD1mon and G85R-hSOD1 are co-expressed (Figure 7B). The images shown in Figure 7A are representative of multiple experiments, which were quantified to confirm the effect of WT monomerized SOD1 of either species on the aggregation of mutant SOD1 of either species (Figure 7B). At 24 hours post-transfection, the cells co-expressing mutant SOD1 of either species with WT-SOD1mon of either species accumulated less insoluble mutant SOD1 (Figure 7B). At 48 hours post-transfection, the suppressive effect of WT-SOD1mon on mutant SOD1 aggregation dissipated except for the combination of WT-hSOD1mon with G86R-mSod1 (Figure 7B). These findings indicate that monomerized WT-hSOD1 or mSod1 retain an ability to interact with misfolded forms of SOD1 that generate detergent insoluble aggregates.

**Figure 8:**
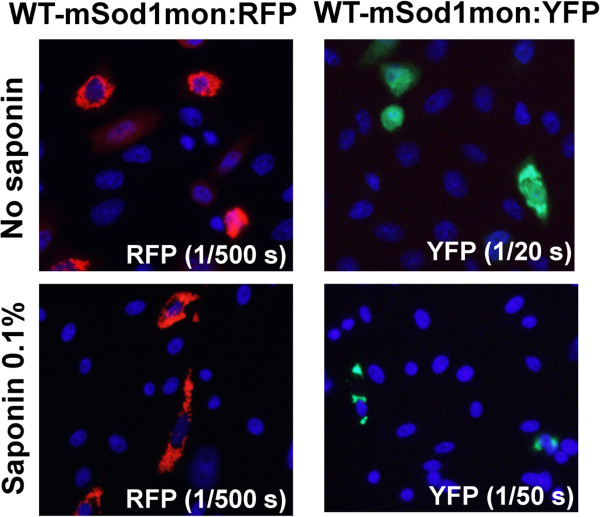
**WT-mSod1mon fused to RFP or YFP forms saponin-resistant inclusions.** The indicated fused constructs were expressed in CHO cells as explained in previous figure legends and Methods.

**Figure 9:**
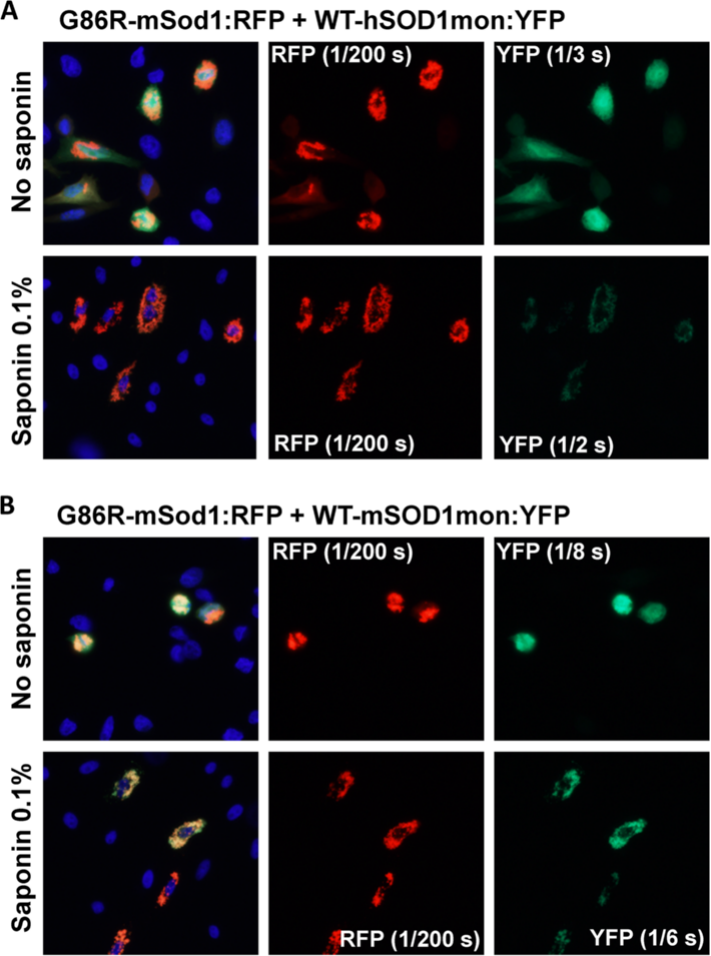
**Co-transfection of G86R-mSod1:RFP with WT-hSOD1mon and WT-mSod1mon fused to YFP. (A** and **B)** The indicated fused constructs were expressed in CHO cells as explained in previous figure legends and Methods. WT-hSOD1mon weakly interacts with G86R-mSod1:RFP inclusions whereas WT-mSod1mon shows a strong saponin resistant interaction.

In our last set of experiments, we used fusion proteins of WT-mSod1mon and WT-hSOD1mon with YFP to investigate further whether monomeric WT protein of either species interacted with misfolded G86R-mSod1. WT-hSOD1mon fusions to RFP or YFP remain soluble and completely releasable by saponin [23]. Unexpectedly, fusions of WT-mSod1mon to either RFP or YFP produced inclusions that were saponin resistant (Figure 8). Additionally, these inclusions were morphologically similar to those produced by mutant hSOD1 fused to either tag. When G86R-mSod1:RFP was co-expressed with WT-hSOD1mon:YFP we observed weak interactions that were only partially resistant to saponin; whereas the interactions between G86R-mSod1:RFP and WT-mSod1mon:YFP were robust and saponin resistant (Figure 9). Thus, the inherently aggregation prone WT-mSod1mon readily co-assembles with inclusions formed by G86R-mSod1:RFP but the more soluble WT-hSOD1mon does not. These findings indicate that monomerization of WT-hSOD1 does not induce it to associate with misfolded G86R-mSod1.

## Discussion

In the present study, we have used cell culture models of mutant SOD1 aggregation to examine interactions between G86R-mSod1 and human SOD1. To conduct these investigations, we used two approaches that offer different ways of detecting SOD1 misfolding. In studies that assess the formation of detergent-insoluble complexes by untagged SOD1, we observed that native hSOD1 does not co-aggregate with G86R-mSod1. In experiments to visualize interactions between misfolded G86R-mSod1 and WT-hSOD1, we observed that as compared to WT-mSod1 fusion proteins, WT-hSOD1 fusion proteins did not readily interact with G86R-mSod1 inclusions. Despite some noted incongruities in the data as described below (summarized in Table 1), these findings are consistent with the idea that misfolded mutant mSod1 has a low potential to template the misfolding of WT-hSOD1 and thus have implications in interpreting prior studies that demonstrated high levels of WT-hSOD1 co-expressed with G86R-mSod1 had no effect on disease course in transgenic mice [13].

### WT and mutant SOD1 interactions within detergent-insoluble aggregates and cellular inclusions

Previous studies of transgenic mice have observed that when WT-hSOD1 proteins were overexpressed with mutant hSOD1, then the age at which mice developed disease phenotypes was dramatically accelerated [7-11]. Importantly, these studies have clearly demonstrated that when WT human SOD1 is co-expressed with fALS mutants of SOD1 that are C-terminally truncated, then WT protein can be detected in detergent-insoluble complexes as co-sedimenting with misfolded mutant SOD1 [7,11]. Overall, these studies suggest that mutant SOD1 may be able to template misfolding of WT-hSOD1, inducing the latter protein to acquire the misfolded conformation of mutant SOD1.

In cell culture models of mutant SOD1 aggregation, we have previously demonstrated that co-expression of WT-hSOD1 transiently inhibits the aggregation of G85R-hSOD1 (24 hours following transfection) but ultimately, at 48 hours after transfection, co-sedimentation of WT-hSOD1 with G85R-hSOD1 in detergent-insoluble complexes was observed [19]. In co-transfections of G85R-hSOD1 with WT-mSod1 we could not discern whether WT-mSod1 ultimately co-sedimented with G85R-hSOD1 because these proteins essentially co-migrate in SDS-PAGE [19]. However, in co-transfections of WT-mSod1 with other mutants (A4V and G93A of hSOD1) it was very clear that WT-mSod1 did not form co-sedimenting complexes with the human mutant proteins [19]. In the course of our analysis of interactions between human and mouse SOD1 using fluorescent fusion proteins, looking at all possible combinations, we noted that WT-mSod1:YFP did not co-aggregate with G85R-hSOD1:RFP (Additional file 1: Figure S4). Collectively, these studies are consistent with the idea that native WT-mSod1 does not readily interact with misfolded forms of G85R-hSOD1; these findings may provide an explanation for why deleting mSod1 in mice expressing G85R-hSOD1 had no obvious effect on disease course [12].

The present study was prompted by the observation by Audet and colleagues in which co-expression of WT-hSOD1 had no effect on the age at which mice expressing G86R-mSod1 develop paralysis [13]. Using a biochemical assay for aggregation in an established cell model, we found no evidence that native WT-hSOD1 co-sediments with G86R-mSod1 in detergent-insoluble complexes. Similarly, using fluorescently tagged SOD1 constructs to visualize aggregation, we observed that inclusions formed by G86R-mSod1:RFP contain little if any WT-hSOD1:YFP; whereas WT-mSod1:YFP was readily incorporated into these structures. Together, these findings are consistent with the idea that native WT-hSOD1 has a low propensity to be induced to misfold by G86R.

However, the strength of the data from examination of RFP and YFP tagged variants of SOD1 is undermined by a number of incongruent observations (Table 1). The primary undermining observation is that WT-hSOD1 fused to YFP does not robustly co-deposit with inclusions formed by G85R-hSOD1 fused to RFP [23] (Table 1). Based on co-transfection studies of untagged WT and G85R-hSOD1 [19], we would have predicted WT-hSOD1:YFP would co-aggregate with G85R-hSOD1:RFP. This incongruity may be discounted by our prior work that has demonstrated that the generation of detergent insoluble complexes does not necessarily equate to inclusion formation [15]. Another troublesome observation was that inclusions formed by G86R-mSod1:YFP could capture monomeric variants of WT hSOD1 fused to RFP (Table 1; Additional file 1: Figure S5). WT-hSOD1mon:RFP on its own appears as a diffusely distributed protein that is released by saponin, but does associate with inclusions formed by mutant hSOD1 fused to YFP including the G85R mutant of hSOD1 [23]. In multiple combinations of co-expression of fluorescently tagged proteins (Table 1), the G85R variant of hSOD1 and the G86R variant of mSod1 show similar abilities to interact with WT human and mouse SOD1 proteins. The main distinguishing feature was that WT-mSod1:YFP readily co-deposited in inclusions formed by G86R-mSod1:RFP, providing a clear example in which homo-specific interactions were favored. Although these incongruities in the data diminish the conclusiveness of this portion of the study, the data overall are consistent with the hypothesis that native WT-hSOD1 does not readily interact with misfolded mutant mouse Sod1.

### Role of dimer formation in mouse and human SOD1 interactions

Experimental conversion of SOD1 from a dimeric to a monomeric enzyme by the mutation of residues 50 (Phe) and 51 (Gly) to Glu was first described by Bertini et al. [25,26]. It is thought that the introduction charged residues at these sites produces a repulsive effect as the two monomers of SOD1 attempt to align as a homodimeric enzymes. These monomeric enzymes retain activity and crystal structures of this experimental variant have demonstrated that the proteins can fold into a near normal conformation [26]. Thus, the engineered monomer of SOD1 is thought to be WT-like in its properties.

To confirm that the mutations that monomerize SOD1 inhibit interactions between subunits, we developed a split-luciferase assay. As predicted, the monomerization mutations in WT-hSOD1 produced a protein that failed to homodimerize. An important consideration in our studies of interactions between mutant SOD1 and monomerized WT-SOD1 was that the mutant variants possessed intact dimer interfaces and thus the effect of the engineered monomer mutations could have been less robust. However, data from the split luciferase assay demonstrated that SOD1 harboring the F50E/G51E mutations did not efficiently dimerize with WT-SOD1. Thus, we are confident that monomerized WT-hSOD1 variants were behaving as monomeric proteins.

Our data demonstrate that, when over-expressed, untagged versions of both WT-hSOD1mon and WT-mSod1mon were prone to form detergent insoluble complexes. Thus, in both the mouse and human proteins, the monomerizing mutations impacted the tendency of these proteins to aggregate. This finding fits with data from several studies that have suggested loss of dimerization is a key step in the aggregation of SOD1 [28-32]. However, the behavior of RFP and YFP tagged monomeric variants of human and mouse SOD1 produced a more complicated picture. We show here that monomeric variants of WT-mSod1 produced saponin resistant inclusions; whereas the monomerized WT-hSOD1:YFP proteins have been shown to exhibit properties of a fully soluble protein [23]. It appears that the mutations that monomerize WT-mSod1 had a more profound impact on structure than the same mutations in WT-hSOD1, inducing the mouse protein to form inclusions. Additionally, it is interesting that the untagged version of WT-hSOD1mon is more prone to adopt detergent insoluble complexes, but a fusion of WT-hSOD1mon to YFP exhibits properties of a soluble protein [23] (Table 1). Notably, we have previously observed that formation of detergent-insoluble complexes does not necessarily equate to formation of inclusions; WT-hSOD1:YFP is generally more prone to lose solubility in non-ionic detergent without producing inclusions [15]. For WT-hSOD1 we conclude that the mutations that monomerize the protein may heighten propensity to aggregate in the presence of non-ionic detergents, but it does seem that the protein is so badly folded that it easily forms the large aggregates that produce visible inclusions.

From transfection of untagged SOD1 variants, we observed that monomerization of WT-hSOD1 had no obvious effect on its interactions or influence over the aggregation of G85R-hSOD1 into detergent-insoluble complexes. Co-expression of WT-hSOD1mon with G85R-hSOD1 produced effects similar to what we have previously reported in co-transfection of native WT-hSOD1 with mutant hSOD1 [19]: initially in 24 hours we observed an inhibition of mutant SOD1 aggregation, but at 48 hours we observe both proteins co-sedimenting in the detergent-insoluble fractions (see Figure 7). Similarly, we observed that WT-mSod1mon transiently inhibited the formation of detergent-insoluble complexes by either G85R-hSOD1 or G86R-mSod1. At 48 hours following transfection, we could clearly discern that WT-hSOD1mon was induced to form co-sedimenting complexes with G85R-hSOD1 (Table 1), but in cells co-expressing WT-hSOD1mon and G86R-mSod1 there was significant reduction in level of aggregated G86R-mSod1 (see Figure 7). For some combinations of co-transfections, it was more difficult to tell whether both proteins co-sedimented because of co-migration in SDS-PAGE. For example, in co-expression of WT-mSod1 with G85R-hSOD1, we were able to confirm the presence of G85R-hSOD1 in the detergent-insoluble fraction using a human specific SOD1 antibody but we were unable to distinguish whether WT-mSod1 was also present (see Figure 7). Overall, in those cases in which we could make definitive observations, our study of the formation of detergent insoluble complexes indicates that monomerization of WT-SOD1 of either species did not abrogate its ability to make homo-specific interactions with mutant SOD1 (mouse to mouse or human to human). This finding indicates normal dimeric interactions are not required to induce the co-aggregation of mutant SOD1 with WT-like protein. Moreover, for both the native fusion proteins of WT-hSOD1or mSod1 and the monomeric variants we observe a preference for homo-specific interactions over hetero-specific interactions.

In our studies of fluorescently-tagged proteins, we observed instances of agreement with our biochemical assays as well as instances of incongruity. In co-expression studies of G86R-mSod1:RFP with WT-hSOD1mon:YFP and WT-mSod1mon:YFP, we observed a preferential recruitment of the WT mouse fusion protein into the inclusions; a finding that corroborated the biochemical assays. However, the apparent selectivity of mouse for mouse was offset by our previous observation that WT-hSOD1mon:YFP did not readily incorporate into inclusions formed by G85R-hSOD1:RFP [23] (Table 1). Moreover, we have observed that WT-hSOD1mon:RFP, which behaves as a soluble protein, readily interacts with inclusions formed by mutant SOD1 fused to YFP [23] (Table 1). WT-hSOD1mon:RFP also interacts with inclusions formed by G86R-mSod1:YFP (Additional file 1: Figure S5); a finding that is somewhat incongruent with our biochemical data. Thus, overall, the data generated by studies with fluorescently tagged variants of human and mouse SOD1 partially corroborate the biochemical.

## Conclusions

In the present study, we have used both biochemical and visual assays to determine the degree to which a variant of mouse SOD1 carrying the fALS mutation (G85R/G86R) interacts with WT-hSOD1. We have also examined the role of normal dimeric interactions between SOD1 subunits in the generation of aggregates that contain both proteins. Our biochemical assays for acquisition of detergent-insolubility, in which untagged SOD1 was over-expressed, indicate that mutant mouse SOD1 preferentially interacts with WT mouse SOD1 and conversely mutant human SOD1 preferentially interacts with WT human SOD1. Mutations that monomerize the WT protein do not significantly diminish these interactions. Thus, for homo-specific interactions in the generation of detergent-insoluble complexes, the normal dimeric interaction is not required. Our visual assays of aggregation and co-aggregation, using YFP tagged proteins, paint a more complex picture possibly because the fluorescent tag that was used to track the protein was not completely benign [23] (Table 1). The most clear-cut observation that could be made was that G86R-mSod1 readily interacts with WT-mSod1 whether native or monomerized. Overall, the data are consistent with the hypothesis that increasing the level of WT-hSOD1 in mice expressing G86R-mSod1 does not accelerate the onset of disease, as observed by Audet et al. [13], because the human protein does not readily interact with misfolded mouse protein.

## Competing interests

The authors declare that they have no competing interests.

## Authors’ contribution

DQ, MP, and KC generated the various YFP and RFP fusion constructs described. DQ carried out the cell imaging experiments. MP carried out the cell transfection and detergent extraction experiments. BR and HB generated luciferase reporter constructs and carried out the experiments. DB designed the experiments and drafted the manuscript. All authors read and approved the final manuscript.

## Supplementary Material

Additional file 1: Figure S1 WT-mSod1:RFP forms saponin-resistant inclusions. **Figure S2.** Additional comparative data for WT-mSod1:RFP co-expressed with WTmSod1:YFP, WT-hSOD1:YFP, or G85R-hSOD1:YFP. **Figure S3.** Immunoblot to demonstrate similar levels of expression of SOD1:GLuc fusion proteins. **Figure S4.** WT-mSod1:YFP does not bind to G85R-hSOD1:RFP inclusions. **Figure S5.** WT-hSOD1mon:RFP forms intermingled inclusions with G86R-mSod1:YFP. **Figure S6.** WT-mSod1mon:YFP forms intermingled inclusions with G85R-hSOD1:RFP. Click here for file
