# Supplementary material for: Features of wild-type human SOD1 limit interactions with misfolded aggregates of mouse G86R Sod1
Source: Mol Neurodegener. 2013 Dec 17;8:46. doi: 10.1186/1750-1326-8-46 (PMC3881023; doi:10.1186/1750-1326-8-46)
Supplement: Additional file 1: Figure S1 — WT-mSod1:RFP forms saponin-resistant inclusions. Figure S2. Additional comparative data for WT-mSod1:RFP co-expressed with WTmSod1:YFP, WT-hSOD1:YFP, or G85R-hSOD1:YFP. Figure S3. Immunoblot to demonstrate similar levels of expression of SOD1:GLuc fusion proteins. Figure S4. WT-mSod1:YFP does not bind to G85R-hSOD1:RFP inclusions. Figure S5. WT-hSOD1mon:RFP forms intermingled inclusions with G86R-mSod1:YFP. Figure S6. WT-mSod1mon:YFP forms intermingled inclusions with G85R-hSOD1:RFP. [file 1750-1326-8-46-S1.pdf]

Features of wild-type human SOD1 limit interactions with misfolded aggregates of mouse G86R SOD1

David Qualls*, Mercedes Prudencio*, Brittany L.T. Roberts, Keith Crosby, Hilda Brown, David R. Borchelt

Supplementary figures

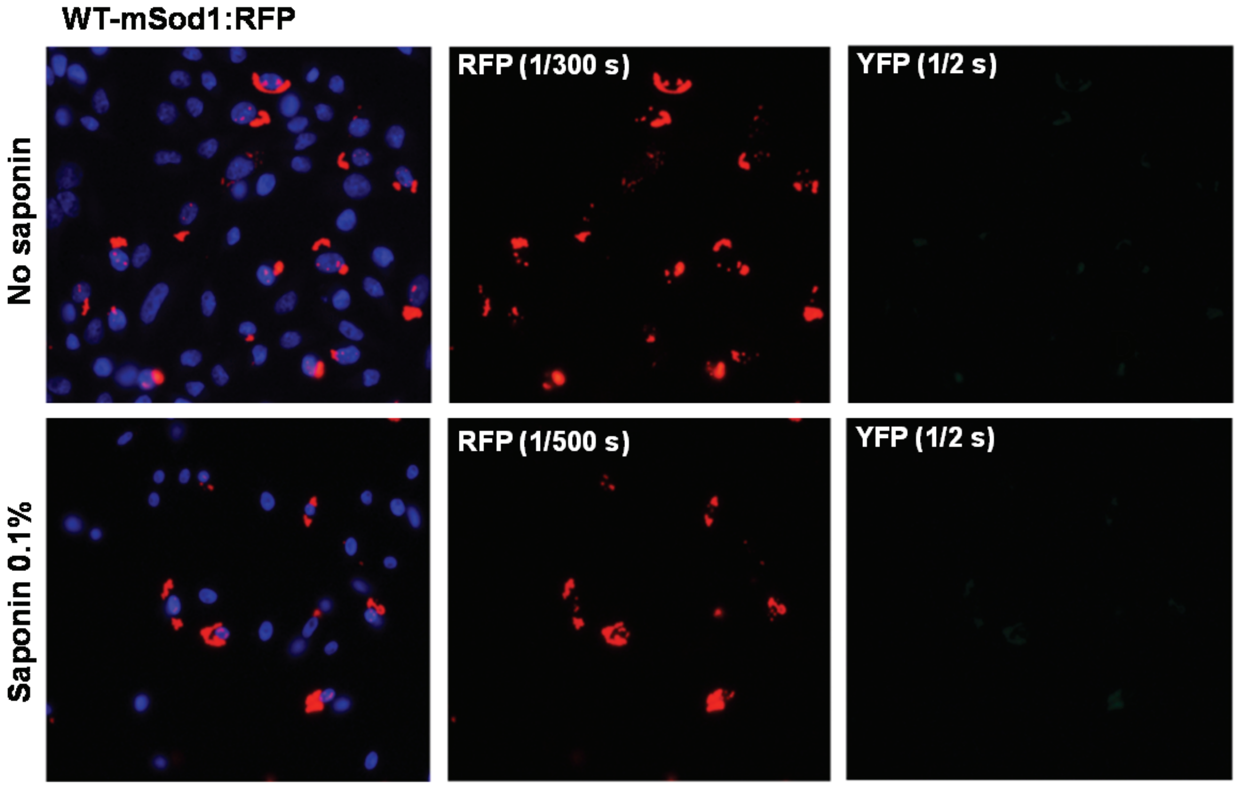

Supplementary Fig. S1. WT-mSod1:RFP forms saponin-resistant inclusions. The indicated fusion construct was expressed in CHO cells as explained in the legends of the primary figures. The images shown are representative of 3 independent experiments.

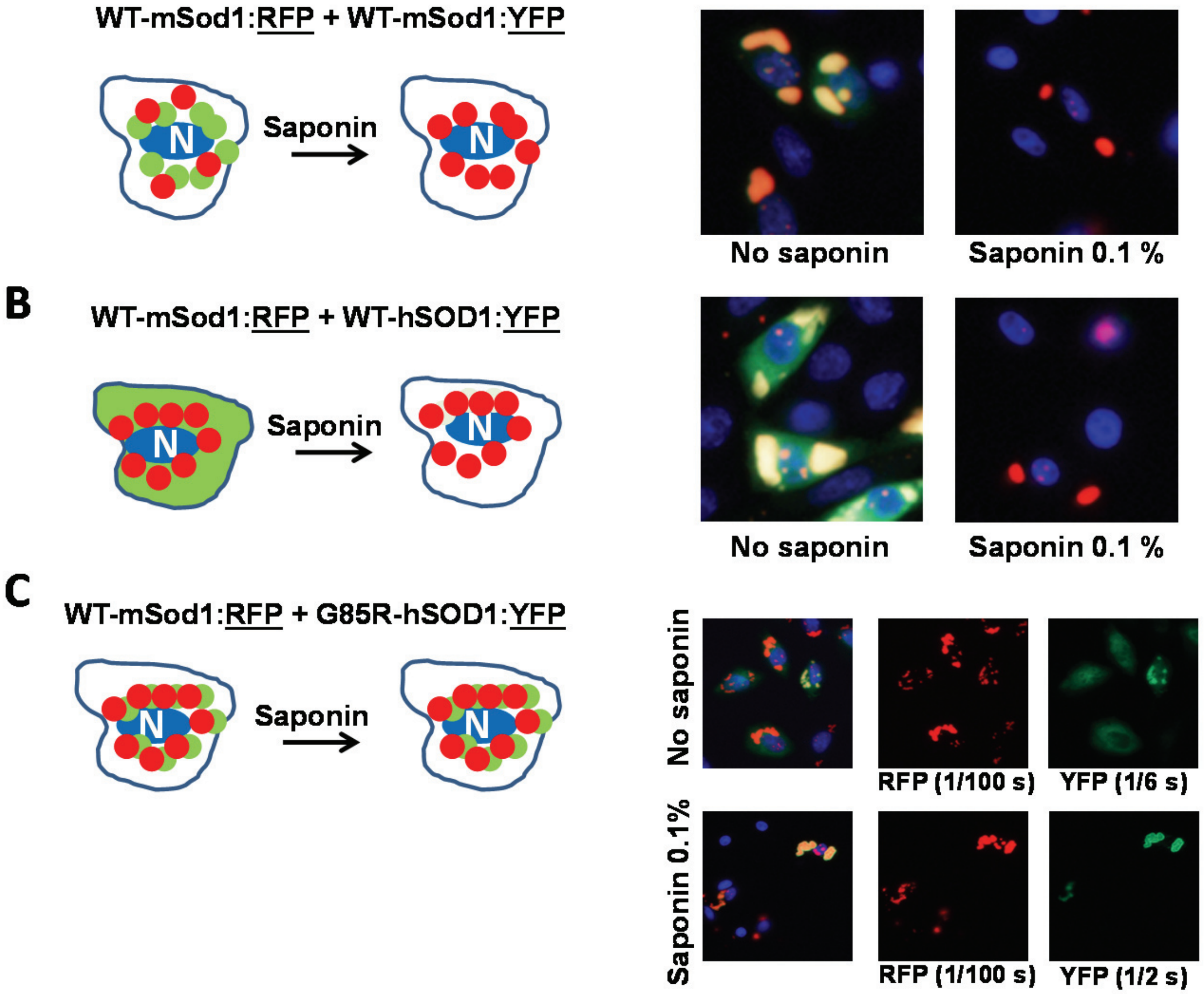

Supplementary Fig. S2. Additional comparative data for WT-mSod1:RFP co-expressed with WT-mSod1:YFP, WT-hSOD1:YFP, or G85R-hSOD1:YFP. The indicated fusion constructs were expressed in CHO cells as explained in the legends of the primary figures. The images shown are representative of 3 independent experiments. The diagrams illustrate the primary observations. WT-mSod1:RFP inclusions do not interact with WT-mSod1:YFP or WT-hSOD1:YFP. G85R-hSOD1:YFP appears to deposit on the surface of the inclusions formed by WT-mSod1:RFP.

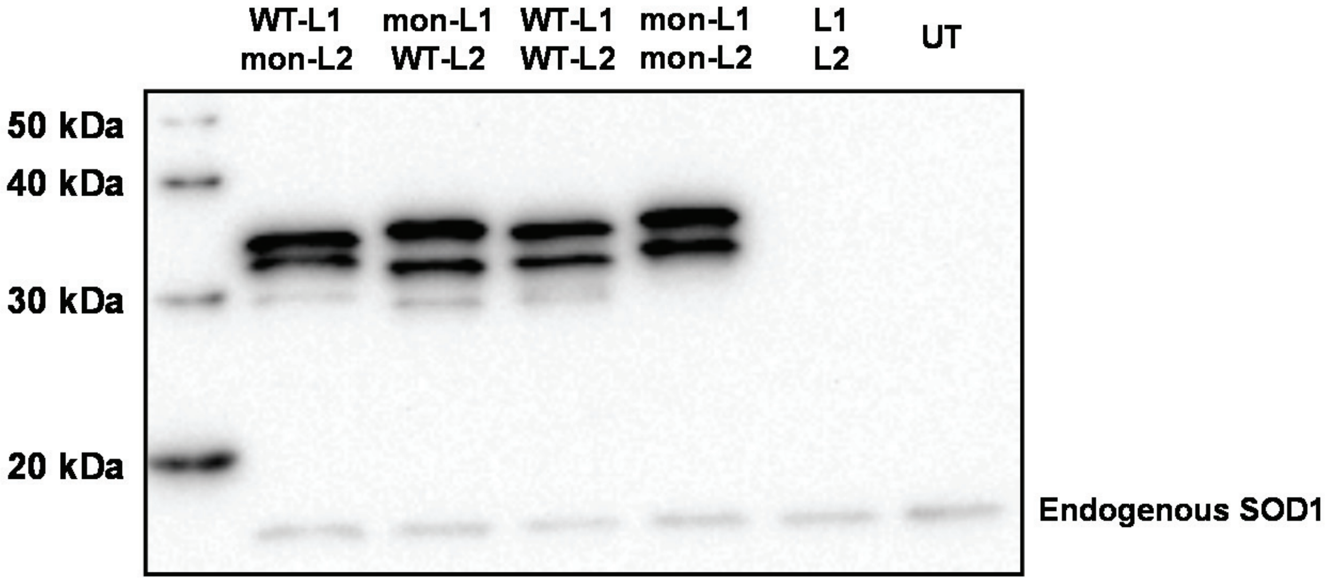

Supplementary Fig. S3. Immunoblot to demonstrate similar levels of expression of SOD1:GLuc fusion proteins. Ten micrograms of protein from freeze-thaw lysates of CHO cells transfected with vectors that express the fusion proteins were analyzed by immunoblot with a rabbit polyclonal antibody raised against the whole human SOD1 protein. These lysates were used in one of the replicates of luciferase assays that generated the data in Figure 6. The identity of each protein in the doublet is provided above the figure; the orientation of the labels matches the orientation of the two bands. UT denotes untransfected cells. The products of the vectors expressing the GLuc fragments cannot be seen with the SOD1 antibody.

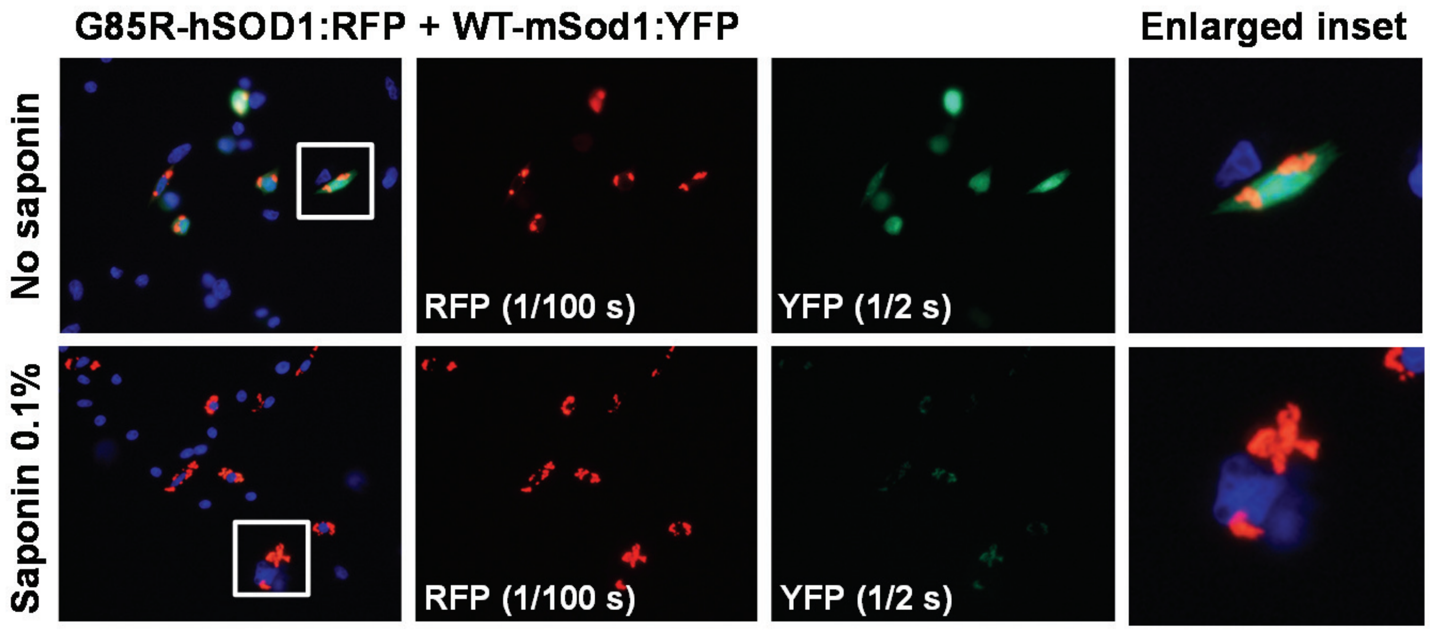

Supplementary Fig. S4. WT-mSod1:YFP does not bind to G85R-hSOD1:RFP inclusions. The indicated fusion constructs were expressed in CHO cells as explained in the legends of the primary figures. The images shown are representative of 3 independent experiments.

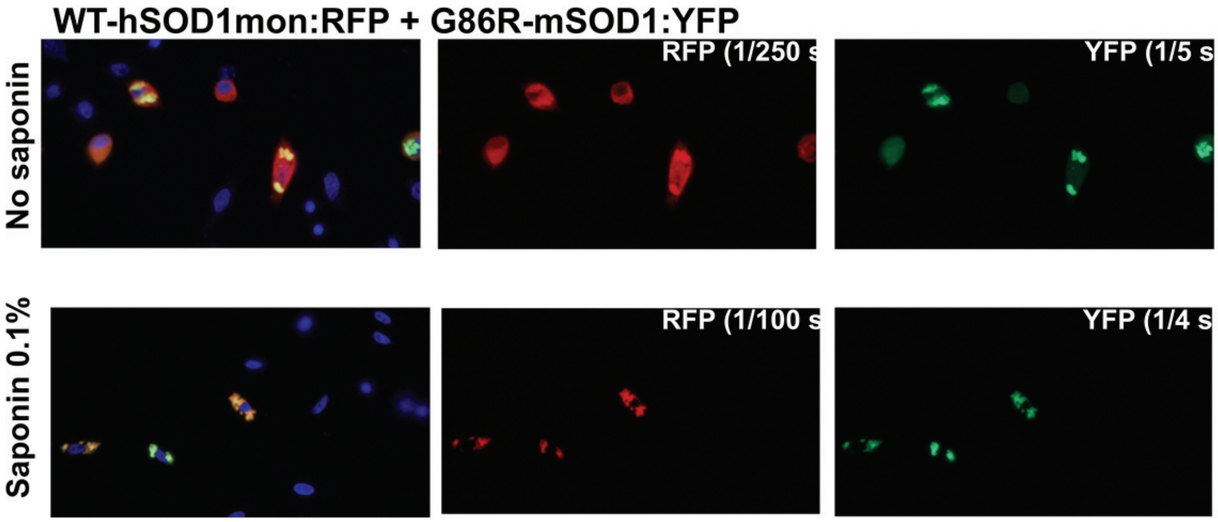

Supplementary Fig. S5. WT-hSOD1mon:RFP forms intermingled inclusions with G86R-mSod1:YFP. The indicated fusion constructs were expressed in CHO cells as explained in the legends of the primary figures. The images shown are representative of 3 independent experiments.

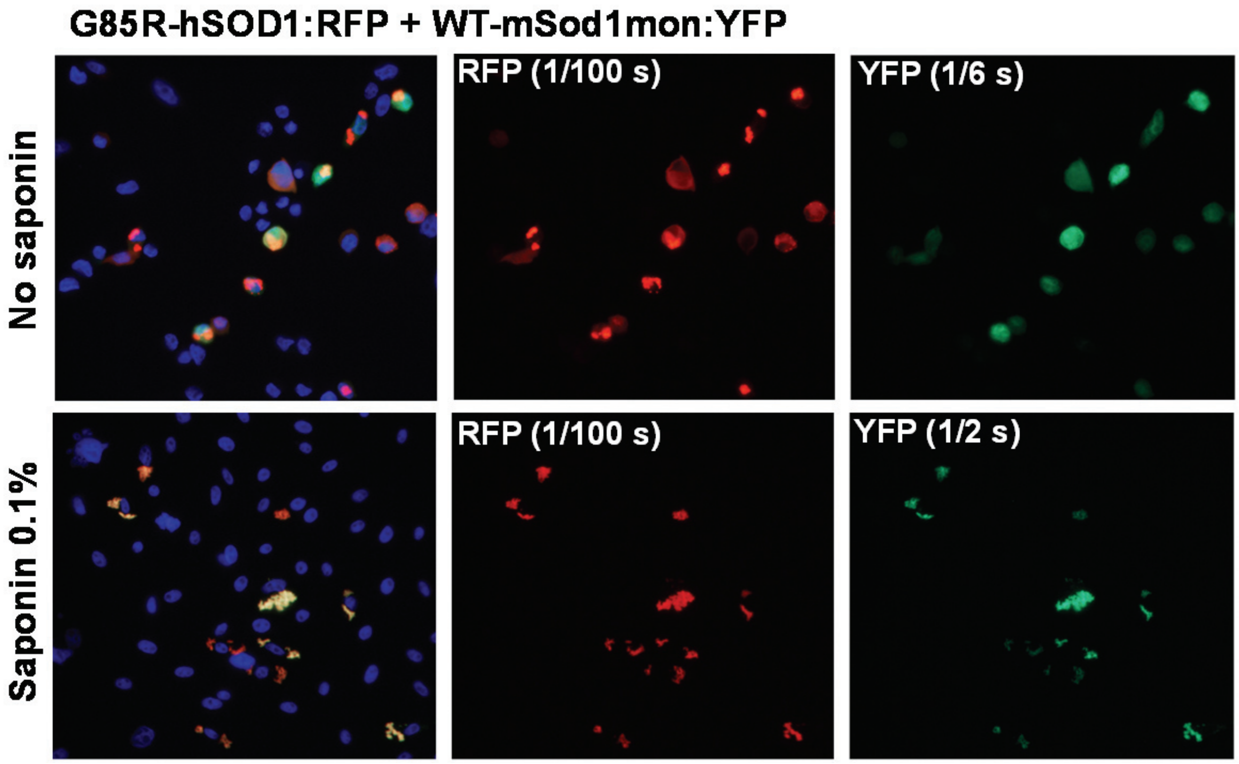

Supplementary Fig. S6. WT-mSod1mon:YFP forms intermingled inclusions with G85R-hSOD1:RFP. The indicated fusion constructs were expressed in CHO cells as explained in the legends of the primary figures. The images shown are representative of 3 independent experiments.
